# *Vital Signs*: Missed Opportunities for Preventing Congenital Syphilis — United States, 2022

**DOI:** 10.15585/mmwr.mm7246e1

**Published:** 2023-11-17

**Authors:** Robert McDonald, Kevin O'Callaghan, Elizabeth Torrone, Lindley Barbee, Jeremy Grey, David Jackson, Kate Woodworth, Emily Olsen, Jennifer Ludovic, Nikki Mayes, Sherry Chen, Rachel Wingard, Michelle Johnson Jones, Fanta Drame, Laura Bachmann, Raul Romaguera, Leandro Mena

**Affiliations:** ^1^Division of STD Prevention, National Center for HIV, Viral Hepatitis, STD, and TB Prevention, CDC; ^2^Division of Birth Defects and Infant Disorders, National Center on Birth Defects and Developmental Disabilities, CDC; ^3^National Center for HIV, Viral Hepatitis, STD, and TB Prevention, CDC.

## Abstract

**Introduction:**

Congenital syphilis cases in the United States increased 755% during 2012–2021. Syphilis during pregnancy can lead to stillbirth, miscarriage, infant death, and maternal and infant morbidity; these outcomes can be prevented through appropriate screening and treatment.

**Methods:**

A cascading framework was used to identify and classify missed opportunities to prevent congenital syphilis among cases reported to CDC in 2022 through the National Notifiable Diseases Surveillance System. Data on testing and treatment during pregnancy and clinical manifestations present in the newborn were used to identify missed opportunities to prevent congenital syphilis.

**Results:**

In 2022, a total of 3,761 cases of congenital syphilis in the United States were reported to CDC, including 231 (6%) stillbirths and 51 (1%) infant deaths. Lack of timely testing and adequate treatment during pregnancy contributed to 88% of cases of congenital syphilis. Testing and treatment gaps were present in the majority of cases across all races, ethnicities, and U.S. Census Bureau regions.

**Conclusions and implications for public health practice:**

Addressing missed opportunities for prevention, primarily timely testing and appropriate treatment of syphilis during pregnancy, is important for reversing congenital syphilis trends in the United States. Implementing tailored strategies addressing missed opportunities at the local and national levels could substantially reduce congenital syphilis.

**Summary**

**What is already known about this topic?**

Since 2012, U.S. congenital syphilis cases increased substantially. Syphilis during pregnancy can lead to stillbirth, miscarriage, infant death, and maternal and infant morbidity, which are preventable through appropriate screening and treatment.

**What is added by this report?**

In 2022, lack of timely testing and adequate treatment contributed to almost 90% of congenital syphilis cases in the United States, including substantial proportions of congenital syphilis cases in all U.S. Census Bureau regions and among all racial and ethnic groups.

**What are the implications for public health practice?**

Implementing tailored strategies addressing missed opportunities at the local and national levels could improve timeliness of testing and appropriateness of treatment for syphilis during pregnancy and thereby reduce the incidence of congenital syphilis and complications of syphilis during pregnancy.

## Introduction

In a time when perinatal infections such as HIV and hepatitis B are declining in the United States (*1*,*2*), cases of congenital syphilis, a disease resulting from perinatal transmission of syphilis, have been increasing substantially. During 2012–2021, the number of reported congenital syphilis cases increased 755%, from 335 during 2012 to 2,865 during 2021 (*3*,*4*). Congenital syphilis can lead to stillbirth, miscarriage, or neonatal death, and surviving infants who are not adequately treated might develop blindness, deafness, developmental delay, or skeletal abnormalities (*5*). Congenital syphilis is preventable through timely testing and adequate treatment of syphilis during pregnancy (*5*). Increases in congenital syphilis mirror trends observed in rates of primary and secondary syphilis cases in women of reproductive age, which increased 676% (from 2.1 to 16.3 per 100,000 population) during 2012–2021 (*4*). Racial and geographic disparities in rates of congenital syphilis and rates of syphilis among women exist (*4*). To reduce perinatal transmission, CDC recommends screening for syphilis during pregnancy at the first prenatal care visit. Where access to prenatal care is not optimal, screening and treatment (if indicated) should be performed as soon as pregnancy is identified (*6*). CDC recommends screening at 28 weeks’ gestation and at delivery for those who 1) live in communities with high rates of syphilis, 2) are at high risk for syphilis acquisition during pregnancy (e.g., substance use or a new sex partner), or 3) were not previously tested during the pregnancy (*6*). Appropriate screening for syphilis during pregnancy, as well as screening of sexually active persons when appropriate, has been shown to prevent syphilis morbidity (*5*,*6*). Identifying missed opportunities (e.g., lack of screening and inadequate treatment) to prevent congenital syphilis and treat syphilis during pregnancy is critical to understanding drivers of the current congenital syphilis surge and to better direct public health interventions (*7*,*8*).

## Methods

### Study Population

Cases of congenital syphilis that meet the 2018 Council of State and Territorial Epidemiologists congenital syphilis case definition* are reported to CDC’s National Notifiable Diseases Surveillance System (NNDSS). Data are from all 50 states, the District of Columbia, and U.S. territories and freely associated states.

### Classification of Missed Opportunities

To identify potential missed prevention opportunities among congenital syphilis-associated pregnancies, a mutually exclusive six-part cascading framework of risk factors was developed that includes 1) no documented testing or nontimely testing, 2) late identification of seroconversion during pregnancy, 3) no treatment or nondocumented treatment, 4) inadequate treatment, 5) clinical evidence of congenital syphilis despite documentation of adequate maternal treatment, and 6) insufficient data to identify a missed prevention opportunity for the case. Using a stepwise approach, cases of congenital syphilis reported via NNDSS in 2022 were examined and assigned to one of the six framework categories, starting with determining whether timely testing occurred during pregnancy, defined as testing completed ≥30 days before delivery (*9*). Cases for which documentation of timely testing was absent were categorized as “nontimely or no documented testing.” Cases for which the syphilis diagnosis was received late in pregnancy (<30 days before delivery), after earlier nonreactive testing (i.e., testing without evidence of syphilis), were categorized as late identification of seroconversion. Congenital syphilis cases for which timely testing led to a syphilis diagnosis during pregnancy were assessed based on whether treatment adequate to prevent congenital syphilis, defined as a penicillin-based regimen initiated ≥30 days before delivery, with dosing and spacing appropriate for the stage of syphilis (*5*,*6*), was documented. Cases without adequate documentation of treatment were categorized as either 1) inadequate treatment or 2) no or nondocumented treatment. Finally, those congenital syphilis cases that occurred despite documentation of timely testing and adequate treatment were categorized as either 1) clinical evidence of congenital syphilis despite adequate treatment during pregnancy or 2) insufficient data to identify the missed opportunity despite careful review.

### Data Analysis

Numbers of congenital syphilis cases and rates of primary and secondary syphilis among females aged 15–44 years in 2022 were compared with annual data from 2012 through 2021. Missed opportunities for prevention were stratified by U.S. Census Bureau region and by race and ethnicity of the birth parent. Prenatal testing and treatment status were stratified according to whether at least one prenatal care visit had occurred during the pregnancy. Analyses were completed using Stata statistical software (version 15.1; StataCorp). This activity was reviewed by CDC, deemed not research, and was conducted consistent with applicable federal law and CDC policy.^†^

## Results

### Congenital Syphilis Cases and Outcomes

**FIGURE 1:**
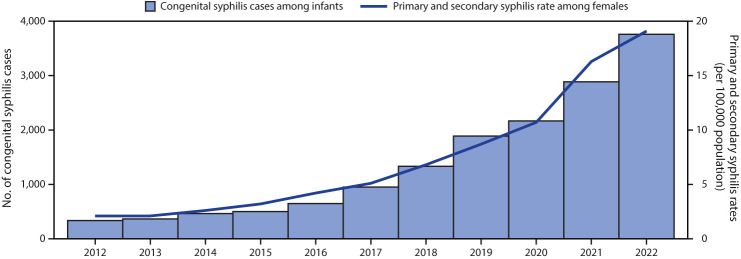
Reported number of cases of congenital syphilis among infants, by year of birth, and rates* of reported cases of primary and secondary syphilis^†^ among females aged 15–44 years, by year — United States, 2012–2022 * Cases per 100,000 population. **^†^** Primary and secondary syphilis case data for all U.S. territories and freely associated states and outlying areas were not available for all years; therefore, rates presented include only the 50 states and the District of Columbia.

In 2022, a total of 3,761 congenital syphilis cases were reported via NNDSS, including 231 (6%) stillbirths and 3,530 (84%) liveborn infants (with 51 [1%] infant deaths). This represents a 31.7% increase in congenital syphilis cases from those reported during 2021, concurrent with a 17.2% increase in rates of primary and secondary syphilis cases among females aged 15–44 years (from 16.3 to 19.1 per 100,000 population) (Figure 1). More than 10 times as many congenital syphilis cases were reported in 2022 (3,761) than in 2012 (334).

### Missed Opportunities for Prevention of Congenital Syphilis

**FIGURE 2:**
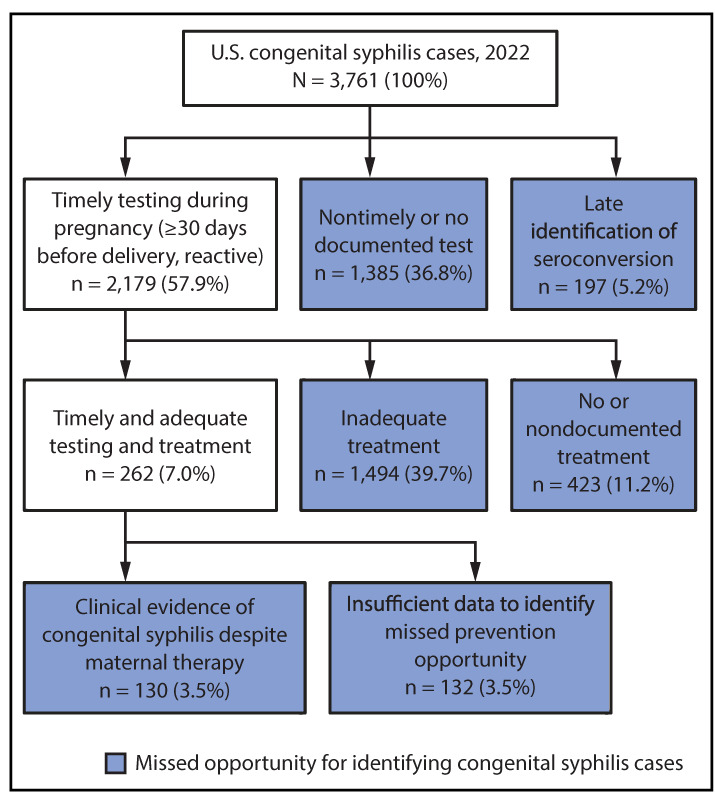
Distribution of congenital syphilis cases, by missed prevention opportunities*^,^^†^^,^**^§^** — United States, 2022 * Timely testing is performed ≥30 days before delivery. ^†^ Late identification of seroconversion is a new reactive syphilis test <30 days before delivery after a nonreactive test earlier in pregnancy. ^§^ Adequate treatment is receipt of a penicillin-based regimen, dosed and spaced appropriately for the stage of syphilis, and commenced ≥30 days before delivery.

Among all (3,761) congenital syphilis cases reported in 2022, the birth parent of most patients (3,302; 87.8%) received either no or nontimely testing (1,385; 36.8%), or no or nondocumented (423; 11.2%) or inadequate (1,494; 39.7%) treatment during pregnancy. Among 197 (5.2%) congenital syphilis cases, syphilis was diagnosed late in pregnancy, after earlier nonreactive testing (Figure 2). Among 2,179 (57.9%) cases for which timely testing and no late identification of syphilis had occurred, more than two thirds (1,494; 39.7% of all congenital syphilis cases) had documentation of inadequate treatment during pregnancy, nearly 20% (423; 19.4% [11.2% of all cases]) received no treatment or nondocumented treatment, and the remaining 262 (12.0% [7.0% of all cases]) received adequate treatment. Among these 262 cases, clinical evidence of congenital syphilis (e.g., on the basis of physical exam, radiographic findings, or laboratory findings) was noted in the newborn despite documentation of adequate treatment in one half (130; 3.5% of all cases), and insufficient data were available to identify missed opportunities to prevent congenital syphilis in the remaining patients (132; 3.5% of all cases).

### Geographic, Racial, and Ethnic Differences in Missed Congenital Syphilis Prevention Opportunities

No testing or nontimely testing accounted for approximately one half of cases in the West (56.2%) and Northeast (50.0%) U.S. Census Bureau regions,^§^ and for the largest percentage of cases in the Midwest region (40.4%). Inadequate treatment accounted for the majority of missed opportunities in the South region (54.5%). No testing or nontimely testing resulted in the highest percentage of missed opportunities for prevention among non-Hispanic American Indian or Alaska Native (47.4%), non-Hispanic Native Hawaiian or other Pacific Islander (61.0%), and non-Hispanic White (40.8%) birth parents. Inadequate treatment was the most prevalent cause for missed prevention opportunities among non-Hispanic Black or African American (39.2%) and Hispanic or Latino (47.4%) birth parents (Table 1).

**TABLE 1:** Prenatal syphilis testing and treatment among birth parents of infants with congenital syphilis, by U.S. Census Bureau region, and by race and ethnicity — United States, 2022

Characteristic	Missed opportunities to prevent CS, no. (%)						
	Testing		Treatment		Outcome		Total
	None or nontimely*	Late identification of seroconversion^†^	Inadequate	None or nondocumented	Clinical evidence of CS despite adequate^§^ prenatal treatment	Insufficient data to identify the missed opportunity	
**All cases**	**1,385 (36.8)**	**197 (5.2)**	**1,494 (39.7)**	**423 (11.2)**	**130 (3.5)**	**132 (3.5)**	**3,761**
**U.S. Census Bureau region^¶^**							
Northeast	83 (50.0)	25 (15.1)	26 (15.7)	14 (8.4)	11 (6.6)	7 (4.2)	**166**
Midwest	182 (40.4)	25 (5.5)	140 (31.0)	58 (12.9)	19 (4.2)	27 (6.0)	**451**
South	469 (23.7)	101 (5.1)	1,080 (54.5)	200 (10.1)	74 (3.7)	57 (2.9)	**1,981**
West	650 (56.2)	45 (3.9)	246 (21.3)	150 (13.0)	25 (2.2)	41 (3.5)	**1,157**
U.S. territories and freely associated states	1 (16.7)	1 (16.7)	2 (33.3)	1 (16.7)	1 (16.7)	0 (—)	**6**
Race and ethnicity**^,††^							
AI/AN	81 (47.4)	7 (4.1)	40 (23.4)	27 (15.8)	8 (4.7)	8 (4.7)	**171**
Asian	9 (39.1)	2 (8.7)	8 (34.8)	1 (4.3)	2 (8.7)	1 (4.3)	**23**
Black or African American	353 (31.5)	80 (7.1)	440 (39.2)	153 (13.6)	53 (4.7)	43 (3.8)	**1,122**
NH/OPI	25 (61.0)	1 (2.4)	10 (24.4)	3 (7.3)	0 (—)	2 (4.9)	**41**
White	422 (40.8)	39 (3.8)	370 (35.8)	126 (12.2)	39 (3.8)	38 (3.7)	**1,034**
Hispanic or Latino	384 (34.8)	56 (5.1)	523 (47.4)	89 (8.1)	20 (1.8)	32 (2.9)	**1,104**
Multiracial	29 (42.0)	3 (4.3)	22 (31.9)	10 (14.5)	3 (4.3)	2 (2.9)	**69**
Other	15 (30.6)	4 (8.2)	22 (44.9)	5 (10.2)	1 (2.0)	2 (4.1)	**49**
Unknown	67 (45.3)	5 (3.4)	59 (39.9)	9 (6.1)	4 (2.7)	4 (2.7)	**148**

**Abbreviations:** AI/AN = American Indian or Alaska Native; CS = congenital syphilis; NH/OPI = Native Hawaiian or other Pacific Islander.

* Timely testing is performed ≥30 days before delivery.

^†^ A new reactive syphilis test <30 days before delivery after a nonreactive test earlier in pregnancy.

^§^ Receipt of a penicillin-based regimen, dosed and spaced appropriately for the stage of syphilis, and commenced ≥30 days before delivery.

^¶^ https://www2.census.gov/geo/pdfs/maps-data/maps/reference/us_regdiv.pdf

** Race and ethnicity of the birth parent.

^††^ Persons of Hispanic or Latino (Hispanic) origin might be of any race but are categorized as Hispanic; all racial groups are non-Hispanic.

Among pregnancies resulting in a congenital syphilis outcome, no prenatal care was documented in 1,426 cases (37.9%). Of the 2,179 cases in which a timely test was obtained during pregnancy, no prenatal care was documented in 445 (20.4%) (Table 2). Among the 1,385 cases of congenital syphilis for which no test or a nontimely test was recorded, no prenatal care was documented for 969 (70.0%).

**TABLE 2:** Receipt of prenatal care among birth parents of infants with congenital syphilis, by prenatal syphilis testing and treatment among those with timely testing* — United States, 2022

Prenatal testing and treatment	Prenatal care, no. (%)	
	None documented	One or more prenatal care visit
**Testing**		
No test or nontimely test	969 (70.0)	416 (30.0)
Late identification of seroconversion^†^	12 (6.1)	185 (93.9)
Timely test* during pregnancy	445 (20.4)	1,734 (79.6)
**Total**	**1,426 (37.9)**	**2,335 (62.1)**
**Treatment among persons who received timely testing**		
No treatment	69 (16.3)	354 (83.7)
Inadequate treatment	362 (24.2)	1,132 (75.8)
Adequate treatment^§^	14 (5.3)	248 (94.7)
**Total**	**445 (20.4)**	**1,734 (79.6)**

* Timely testing is performed ≥30 days before delivery.

^†^ A new reactive syphilis test <30 days before delivery after a nonreactive test earlier in pregnancy.

^§^ Receipt of a penicillin-based regimen, dosed and spaced appropriately for the stage of syphilis, and commenced ≥30 days before delivery.

## Discussion

Lack of timely testing and adequate treatment during pregnancy contributed to 88% of congenital syphilis cases in 2022 and represent missed opportunities to prevent maternal syphilis-associated morbidity. Lack of timely testing and adequate treatment contributed to substantial proportions of cases in all geographic areas and in all racial and ethnic groups. Timely testing without evidence of late seroconversion occurred in 58% of cases; however, inadequate treatment occurred in 69% of these cases, and no treatment or nondocumented treatment in 19%. Treatment could be considered inadequate based on inappropriate selection of an antimicrobial agent, dosing, or spacing of doses, as well as an insufficient interval between initiation of treatment and delivery; ongoing analyses aim to describe specific sources of inadequate treatment to better guide public health action. Strategies that reduce loss to follow-up and decrease the time between testing and treatment could increase the likelihood of adequate treatment. This outcome has been achieved at some medical facilities and health organizations through implementation of rapid syphilis point-of-care testing (*10*), which the World Health Organization recommends during pregnancy in settings where a delay in diagnosis can lead to loss to follow-up (*11*). Innovations in treatment and close follow-up (e.g., field-delivered treatment and disease intervention specialists trained to prevent and control infectious diseases providing linkage to care) can help facilitate adequate treatment (*12*–*14*).

### Recommended Treatment for Prevention of Congenital Syphilis

Benzathine penicillin G is the only recommended treatment for syphilis during pregnancy; this drug must be administered as an injection by a trained professional as either a single dose or as 3 doses spaced 7–9 days apart, depending on the stage of infection (*6*). The success rate of this treatment in preventing congenital syphilis has been reported to be as high as 98% (*15*). Although this analysis includes cases with clinical evidence of congenital syphilis despite adequate treatment, some of these cases might be explained by undetected reinfection late in pregnancy. Because the United States is currently facing a shortage of benzathine penicillin G, CDC has encouraged providers and health departments to prioritize benzathine penicillin G for the treatment of syphilis in pregnancy.*^¶^*

### Individual Screening Based on Risk Factors and Community Syphilis Rates

Historically, syphilis screening and interventions have targeted individual risk factors, but for many sexually active persons, their most significant risk factor is living in a community with high rates of syphilis (*4*,*6*). CDC guidelines recommend syphilis screening for sexually active persons in communities with high rates of syphilis (*6*); however, the threshold for a high rate is not defined. Currently, the Healthy People 2030 goal is to reduce the rate of primary and secondary syphilis cases among females aged 15–44 years to 4.6 per 100,000 population.** In counties with a rate that exceeds this goal, offering syphilis testing to sexually active females aged 15–44 years and their sex partners might help identify syphilis cases and prevent spread, support progress toward meeting the Healthy People 2030 goals, and reduce congenital syphilis. In 2021, 38% of U.S. counties, accounting for 72% of the U.S. population, had syphilis rates above the goal level^††^ . Disparities in syphilis rates by race and ethnicity are not explained by differences in sexual behaviors, but rather reflect access to sexual health care, differences in sexual networks, and persistent and systemic racism in medical care (*6*,*16*). Screening based on geographic risk can decrease stigma and biases associated with screening based on individual risk factors. In counties already at or below the Healthy People 2030 goal level, clinicians should continue to assess individual risk factors (e.g., diagnosis of other sexually transmitted infections, a new partner, history of incarceration, transactional sex work, or being a male aged <29 years) to determine screening needs.^§§^

More than 37% of infants with congenital syphilis were born to persons who had received no prenatal care. Among congenital syphilis cases, no or nontimely testing during pregnancy was the most frequently missed opportunity identified among birth parents without documented prenatal care. Among those with a timely test obtained during pregnancy, 20.4% had no prenatal care documented, suggesting that testing occurred outside prenatal care. In addition to improving access to prenatal care, approaches to providing care outside of clinical settings (e.g., use of rapid tests, field-delivered treatment, active case follow-up, and linkage to care by disease intervention specialists) are needed to ensure appropriate and timely screening and treatment. Any encounter with medical or public health professionals during pregnancy is an opportunity to identify and treat syphilis, thereby preventing congenital syphilis as well as maternal morbidity. Screening for syphilis at encounters outside traditional prenatal care (e.g., emergency department, jail intake, syringe services program, and maternal and child health programs) might help identify and treat persons with syphilis who might not otherwise receive adequate prenatal care (*13*,*14*,*17*–*19*). In addition, the identification of syphilis during pregnancy should be seen as a high priority for rapid follow-up, with a systematic approach to defining who will be responsible for ensuring timely treatment.

### Limitations

The findings in this report are subject to at least three limitations. First, national congenital syphilis case data contain limited information about social determinants of health. The underlying individual and structural barriers (e.g., systemic inequities and limited health care access) leading to the missed opportunities described in this report are beyond the scope of this analysis. Second, jurisdictional differences in reporting completeness and accuracy for congenital syphilis cases likely exist, including differing legal requirements for screening. Differential reporting might have resulted in misclassification of the missed opportunities, amplifying regional differences. Finally, national case data provide limited information on the breadth of syphilis testing during pregnancy (e.g., prepregnancy testing and the titers of syphilis tests measured during pregnancy), which might lead to misclassification both in the context of a history of adequately treated syphilis, as well as seroconversion late in pregnancy. Testing and treatment that occurred but are not documented cannot be assessed.

### Implications for Public Health Practice

Congenital syphilis rates are rapidly increasing in the United States and are at the highest level in at least 30 years (*4*). Barriers to congenital syphilis prevention are multifactorial, including those at the patient level, such as substance use and insurance status, and those at the system level, such as structural inequities, limited access to health care, and medication shortages (*5*,*8*,*16*,*17*,*20*). Addressing patient and system-level barriers to accessing testing, treatment, and care could help prevent congenital syphilis. Improvements in timely testing and appropriate treatment of syphilis through tailored strategies at local and national levels will help control the congenital syphilis epidemic in the United States.

## Footnotes

*
https://ndc.services.cdc.gov/case-definitions/syphilis-2018/

† 45 C.F.R. part 46.102(l)(2), 21 C.F.R. part 56; 42 U.S.C. Sect. 241(d); 5 U.S.C. Sect. 552a; 44 U.S.C. Sect. 3501 et seq.

§
https://www2.census.gov/geo/pdfs/maps-data/maps/reference/us_regdiv.pdf

¶
www.cdc.gov/std/dstdp/dcl/2023-july-20-Mena-BicillinLA.htm

**
https://health.gov/healthypeople/objectives-and-data/browse-objectives/sexually-transmitted-infections/reduce-syphilis-rate-females-sti-03

††
https://www.cdc.gov/nchhstp/atlas/syphilis/index.html

§§
https://www.cdc.gov/std/treatment-guidelines/screening-recommendations.htm
